# Triggers of acute attacks of gout, does age of gout onset matter? A primary care based cross-sectional study

**DOI:** 10.1371/journal.pone.0186096

**Published:** 2017-10-12

**Authors:** Abhishek Abhishek, Ana M. Valdes, Wendy Jenkins, Weiya Zhang, Michael Doherty

**Affiliations:** Academic Rheumatology, University of Nottingham, Clinical Sciences Building, Nottingham City Hospital, Nottingham, United Kingdom; Leibniz-Institut fur Pflanzengenetik und Kulturpflanzenforschung Gatersleben, GERMANY

## Abstract

**Objectives:**

To determine the proportion of people with gout who self-report triggers of acute attacks; identify the commonly reported triggers, and examine the disease and demographic features associated with self-reporting any trigger(s) of acute attacks of gout.

**Methods:**

Individuals with gout were asked to fill a questionnaire enquiring about triggers that precipitated their acute gout attacks. Binary logistic regression was used to compute odds ratio (OR) and 95% confidence intervals (CI) to examine the association between having ≥1 self-reported trigger of acute gout and disease and demographic risk factors and to adjust for covariates. All statistical analyses were performed using STATA.

**Results:**

550 participants returned completed questionnaires. 206 (37.5%) reported at least one trigger of acute attacks, and less than 5% reported >2 triggers. Only 28.73% participants reported that their most recent gout attack was triggered by dietary or lifestyle risk factors. The most frequently self-reported triggers were alcohol intake (14.18%), red-meat or sea-food consumption (6%), dehydration (4.91%), injury or excess activity (4.91%), and excessively warm or cold weather (4.36% and 5.45%). Patients who had onset of gout before the age of 50 years were significantly more likely to identify a trigger for precipitating their acute gout attacks (aOR (95%CI) 1.73 (1.12–2.68) after adjusting for covariates.

**Conclusion:**

Most people with gout do not identify any triggers for acute attacks, and identifiable triggers are more common in those with young onset gout. Less than 20% people self-reported acute gout attacks from conventionally accepted triggers of gout e.g. alcohol, red-meat intake, while c.5% reported novel triggers such as dehydration, injury or physical activity, and weather extremes.

## Introduction

Gout is the commonest inflammatory arthritis [1] and is a direct consequence of hyperuricaemia which results in intra- and/or peri-articular monosodium urate (MSU) crystal deposition [2]. Intermittent episodes of florid acute inflammation are the characteristic presentation of acute gout. The factors that trigger acute gout are under-researched but many drug, dietary and lifestyle factors are implicated, resulting in common advice regarding lifestyle and dietary modifications to prevent gout attacks [3]. While one internet-based case-crossover study identified several dietary and lifestyle triggers associated with acute gout [4–8], and a study from New Zealand identified multiple triggers [9], lot of the information on dietary and lifestyle triggers of acute gout attacks is based on anecdotal reports, and the mechanism by which some of them trigger acute gout attacks is unclear. For example, tomatoes, and purine intake which are reported to trigger gout attacks actually raise the serum uric acid (SUA) [9, 10]. Paradoxically, it is a reduction in SUA that is believed to trigger acute gout via increased crystal dissolution which then encourages crystal “shedding” [6, 9]. However, an increase in SUA could trigger acute attacks as UA is a pro-inflammatory chemical [11]. Additionally, it is possible that some dietary factors e.g. bioactive lipids can trigger gout attacks by stimulating toll like receptors which then activate the NALP-3 inflammasome [12], which plays a central role in MSU crystal induced inflammation [13].

Thus, the overall purpose of this study was to identify the self-reported triggers of acute gout in community-derived people with gout. The specific objectives were to determine: [1] the proportion of people with gout who identify triggers of acute attacks; [2] the commonly reported triggers, and [3] whether disease and demographic characteristics of people who self-report possible triggers of acute gout differs from those who do not recognise any triggers.

## Methods

This was a cross-sectional study. Participants attending for the final visit of a 2-year community-based randomized clinical trial of nurse-led versus GP-led treatment of gout (n = 516) between 2014 and 2016 were given a questionnaire asking them to identify triggers of gout. This questionnaire was also mailed to 150 of the 222 people with gout who previously participated in a 1-year community-based proof-of-concept study of nurse-led treatment of gout (n = 110), or in the Nottingham gout osteoarthritis biomarker study (n = 112) at Academic Rheumatology, University of Nottingham, UK, and had consented to be contacted regarding future gout research, and were alive in 2015 [14–16]. All participants met the preliminary 1977 American Rheumatism Association classification criteria for gout [17], and the studies were approved by the Nottinghamshire Research Ethics Committee 1. All participants in the randomized controlled trial gave written informed consent. Completion of the mailed questionnaire was implied consent for those who responded to the postal survey. Participants of the 1-year community-based proof-of-concept study of nurse-led treatment of gout and the Nottingham gout osteoarthritis biomarker study who responded to the questionnaire were invited for a single study visit at which blood was collected for SUA measurement, and height and weight were measured after obtaining written informed consent [15, 16]. The randomized controlled study participants had blood collected and anthropometric measurements at their last study visit. All participants self-reported information about physician diagnosed cardiovascular comorbidities e.g. diabetes, hypercholesterolemia, hypertension, heart disease, stroke, and about renal failure.

The questionnaire asked participants to identify all factors that triggered their acute gout attacks using an open question encouraging spontaneous reporting of triggers. Additionally, they were asked whether cold or hot weather triggered their gout attack, and were asked to identify whether their most-recent gout attack was triggered by specific factors such as undertaking more than usual physical activity, injury to the index joint, eating or drinking alcohol excessively, greater than usual intake of red-meats, and drinking less non-alcoholic liquids than usual in the preceding 1–2 days (See S1 File).

### Statistical analysis

Mean ± standard deviation (SD) and number (percentage) were used for descriptive purposes. Items and activities that were self-reported as being perceived triggers of acute gout were classified into groups by a senior research nurse and a rheumatologist (WJ, AA). Independent sample t-test, Kruskal-Wallis test and chi-square tests were used to compare continuous and categorical variables respectively. Cardiovascular comorbidity score was calculated by adding the number of cardiovascular comorbidities (range 0 to 5). Odds ratio (OR) and 95% confidence interval (95% CI) were calculated to examine the association between having any (i.e. ≥1) identified self-reported triggers of acute gout and current age (tertiles), sex (female 0, male 1), body mass index (BMI) (tertiles), tophi (absent 0, present 1), age of onset of gout (<50 years, 50–59 years, and ≥60 years), urate lowering treatment (no 0, yes 1), cardiovascular comorbidity score (0,1,>1) and study recruited from (randomized clinical trial 0, postal survey of previous study participants 1) with no self-reported triggers of acute gout as referent. Binary logistic regression was used to adjust the ORs for all covariates included.

### Sensitivity analysis

As the recollection of risk factors for triggers of acute gout may be affected by biased recall, the analysis was repeated after excluding those older than 80 years in age who may have cognitive decline or long disease duration making it difficult to self-report triggers of acute gout. P <0.05 by 2-sided test were considered statistically significant. All analyses were performed using STATA version 14.

## Results

550 community-derived participants from the East-Midland region (424 of 516 participants in the RCT, and 126 of 150 people who participated in previous observational studies and consented to be contacted again regarding gout research) completed the questionnaires. Their mean (SD) age, disease duration, age of onset of gout, BMI, cardiovascular comorbidity score and SUA were 65.77 (10.78) years, 15.11 (10.79) years, 50.54 (13.57) years, 29.73 (5.05) kg/m^2^, 1.24 (1.17) and 324.81 (118.84) μmol/L respectively. There were 9.3% women, 4.9% had tophaceous gout, and 79.1% were on urate lowering treatment (ULT) when surveyed.

206 (37.5%) participants reported ≥1 trigger of acute gout attack, but very few reported multiple triggers (Table 1). The most frequently self-reported triggers were alcohol (14.2%), dehydration (4.9%), injury or excess activity (4.9%), excessively warm or cold weather (4.4% and 5.5% respectively), and red-meat (4.4%). Less frequently identified triggers were fruits or fruit juices (2.6%), sea-food (2.2%), cheese or cream (2.0%), Chinese sauces or curry (1.6%), vegetables (1.3%), air travel (1.3%), stress (1.1%), diuretics (0.6%), tiredness (0.6%), infection (0.4%) and holiday (0.4%), overindulgence (0.4%).

**Table 1:** Number of triggers of acute gout identified by gout patients.

Number of triggers	% participants reporting
0	62.6%
1	26.0%
2	8.6%
3	20%
4	0.6%
5	0.4%

Patients reporting ≥1 triggers had greater number of acute gout attacks in the previous 12 months compared to those who reported no perceived triggers (median 2, interquartile range 1–4 vs. median 2, interquartile range 1–3, p = 0.04, Kruskal-Wallis test) and had similar disease duration (mean (SD) 15.31 (10.26) years versus 14.99 (11.11) years, p = 0.74). However, responders who identified ≥1 triggers were significantly less likely to have onset of gout after the age of 50 years (Table 2).

**Table 2:** Association between disease and demographic factors and self-reported triggers of gout.

	self-reported triggers		OR (95%CI)	aOR (95%CI)^1^
	absent	present		
Age (years, tertiles)				
1 (≤61.78)	92	85	1	1
2 (61.98–70.45)	109	68	0.68(0.44–1.03)	0.91 (0.56–1.48)
3 (≥70.49)	128	48	0.41 (0.26–0.63)	0.70 (0.38–1.30)
Sex				
female	36	15	1	1
male	308	191	1.49 (0.79–2.79)	1.01 (0.51–2.01)
BMI (Kg/m^2^, tertiles)				
1 (≤27.34)	104	71	1	1
2 (27.33–30.85)	109	66	0.89 (0.58–1.36)	0.96 (0.61–1.52)
3 (≥30.86)	113	62	0.80 (0.52–1.24)	0.92 (0.58–1.47)
Tophi				
No	312	188	1	1
Yes	14	12	1.42 (0.64–3.14)	1.20 (0.51–2.81)
Age of onset, years				
<50	128	112	1	1
50–59	90	46	0.58 (0.38–0.90)	0.62 (0.38–0.99)
≥60	115	40	0.40 (0.26–0.62)	0.51 (0.29–0.91)
Serum uric acid, (μmol/L, tertiles)				
1 (≤250)	115	60	1	1
2 (251–359)	105	70	1.28 (0.83–1.97)	1.48 (0.93–2.35)
3 (≥360)	105	70	1.28 (0.83–1.97)	1.30 (0.73–2.30)
Urate lowering treatment				
no	68	42	1	1
Yes	258	158	0.99 (0.64–1.53)	1.09 (0.59–1.99)
Cardiovascular comorbidity score				
0	102	79	1	1
1	109	60	0.71 (0.46–1.09)	1.03 (0.63–1.67)
≥2	133	67	0.65 (0.43–0.99)	0.95(0.58–1.57)

^**1**^Data from 509 participants was included in the adjusted analysis as data were missing for age, BMI, age of onset of gout, SUA, tophi, and ULT on 20, 25, 19, 25,24, and 24 participants respectively.

28.7% of responders reported that their most recent gout attack was triggered by a pre-specified dietary or lifestyle factor. The proportion of participants who reported that their most recent acute gout attack was related to each of the pre-specified risk factors ranged between 4.2% and 14.9% (Table 3).

**Table 3:** Percentage of people with gout who attribute their previous attack to dietary and lifestyle factors.

Dietary/lifestyle triggers	% participants reporting^1^
More than usual alcohol or calorie intake	14.91%
Drink less non-alcoholic liquids	10.18%
More than usual physical activity	6.91%
Eat more red meat*	5.27%
Joint injury	4.18%

*beef, lamb, pork,

^1^data missing for 1 participant

### Sensitivity analysis

On including 479 people younger than 80 years in age, the association between age of onset of gout, and self-reported triggers of acute gout remained unchanged, with gout onset between age 50–60 years, and >60 years associating negatively with ≥1 self-reported trigger of acute gout, with aOR (95%CI) 0.60 (0.37–0.98), and 0.44 (0.23–0.81), with gout onset before the age of 50 years referent.

## Discussion

This is a survey of community-derived people with gout enquiring about modifiable factors that may trigger acute gout attacks. It reports that >60% people with gout do not identify any trigger for their acute attacks and that very few people identify multiple triggers. It also shows that people who present with gout after the age of 50 years are significantly less likely to have an identifiable trigger for their acute gout attacks.

While excess alcohol and purine intake, and cold weather are strongly supported as risk factors for acute attacks of gout [6, 18], this study also supports consideration of dehydration, joint injury or excess physical activity, and both unduly warm and cold weather as triggers of acute gout. Excess physical activity and dehydration both cause hyperuricemia, potentially via increased nucleotide breakdown and via lactic acidosis mediated hypouricosuria [19–21]. Increased temperature may operate through a similar mechanism, by causing dehydration, and highest SUA occurs in the month of July in the Northern hemisphere. This is consistent with the observation that gout attacks most frequently occur in both summer [22] and winter months [23]. These observations raise the possibility that temporary increases in serum uric acid may trigger gout attacks via a pro-inflammatory effect [24], as does abrupt reductions in the SUA via crystal dissolution. Additionally, activation of the NALP-3 inflammasome or toll like receptor by exposure to food items such as saturated fatty acids or alcohol may also trigger gout attacks [25–28].

The findings of this study imply that risk factors that trigger gout attacks are person-specific, and that people with gout should be advised to avoid the factors that they recognise may trigger their gout attacks rather than be unduly concerned by all putative triggers. None of the participants in this study self-reported initiation of ULT as a trigger for acute gout. This may be due to the fact that the majority of participants in this study received slow up-titrated ULT as recommended by the British Society for Rheumatology (BSR) while participating in our two intervention studies [14, 29]. Very small proportion of people self-reported infection as a trigger for acute gout, which is contrary to the anecdotal experience of infection being a common trigger for acute Calcium Pyrophosphate Crystal arthritis. None of the participants self-reported hospitalization as a trigger for acute gout.

There are few published studies of self-reported triggers of acute gout. The proportion of people who self-reported alcohol as a trigger for acute gout in this study was significantly smaller (15% vs 47.1%) to that reported by Flynn et al [9]. Similarly, our study using data from an English cohort demonstrated a significantly lower prevalence of dietary triggers of gout compared to the Flynn et al study from New Zealand [9]. For example 62.5% and 35.2% of the participants self-reported seafood/fish, and red meat respectively as a trigger for acute gout [9], while in our current study the proportion self-reporting seafood/fish or red meat as a trigger was small (c. 3–5%). The reasons for this may include differences in the UK and New Zealand general populations’ diet, lifestyle and their perception of triggers of acute gout. It is of interest that in the New Zealand study, the Maori and other Pacific islanders were significantly more likely to self-report dietary and lifestyle risk factors as triggers of acute gout with OR between 3.87 and 1.91 after adjusting for other covariates, suggesting that these differences may be due to cultural perceptions or significant differences in lifestyle [9]. Additionally, the study from New Zealand enquired about dietary factors that may trigger acute gout, while we asked a broader question encompassing all potential triggers which may have inhibited the recall of dietary triggers of acute gout (S1 File) [9].

Except for joint injury, which may trigger acute gout attacks by destabilising crystal deposits, the mechanism by which other risk factors trigger acute gout attacks are poorly understood. Previous studies have identified polymorphisms in the NALP3 inflammasome, TLR-4, CARD8, IL-1β, IL-18, and CD14 genes to be associated with gout [30–35]. A cross sectional study examining genetic association in European and Maori New Zealanders and Caucasian controls found a significant multiplicative interaction between polymorphisms in the CARD8 and IL-1β genes and gout, further supporting the role of a pro-inflammatory genotype in gout pathogenesis [33]. Thus, further studies are required to understand if these genetic changes explain the propensity for developing recurrent gout attacks, and for gout attacks to be triggered by pro-inflammatory dietary and lifestyle factors.

This study reports that people who develop gout after the age of 50 years are less likely to recognise triggers of acute gout after adjusting for other covariates including comorbidities. This may be due to the fact that the prevalence of dietary and lifestyle risk factors for acute gout e.g. alcohol intake, red-meat or fish intake, physical activity or injury etc. reduces with increasing age. Alternatively, it is possible that people with young onset gout have a more pro-inflammatory genotype wherein a short-lived local or systemic metabolic change induces inflammatory reactions. Further research is required to investigate this hypothesis.

There are several caveats to the study. Firstly, it is possible that the recall of triggers of acute gout may be biased by disease duration. However, this seems unlikely since those who self-reported any gout trigger had comparable disease duration (mean (SD) 14.93 (11.22) vs 15.28 (10.37) years, p = 0.72) compared to those who did not report any triggers. The associations were unchanged when people >80 years in age were excluded from the analysis. Nevertheless, it is possible that the results of this study are affected by biased recall given the long disease duration and retrospective study design that requires self-report of triggers of acute gout. However, this is unlikely to be a significant caveat as similar results were reported for the most recent gout attack. Data from people with gout who participated in three different research studies were included in this analysis. However, all three studies recruited from the patient lists of GP surgeries in and around Nottinghamshire, and had comparable disease and demographic characteristics [16]. Also, since we used open-ended questions to enquire about triggers of acute gout, we did not capture information on the type and amount of alcohol intake that could trigger an acute gout, and such detail requires further study. Additionally, these self-reported gout attacks were not required to meet the American College of Rheumatology classification criteria for acute gout, as these criteria were published after the study was already underway [36]. Finally, all these perceived triggers were self-reported, and further studies are required to confirm the validity of these findings.

In conclusion, most people with gout do not identify any triggers that precipitate their gout attacks, and those that do usually only identify 1–2 triggers. This implies that people with gout should be advised to avoid the specific risk factors that appear to trigger their gout attacks, and not be unduly concerned about all potential triggers.

## Key message

- Most people with gout identify no or only one trigger of acute attacks of gout
- People with young onset gout are more likely to identify such triggers
- Avoidance of multiple factors that are anecdotally reported to trigger gout attacks is inappropriate

## Supporting information

S1 Dataset Anonymised dataset for analysis. (XLS)

S1 File (DOCX)
