# Supplementary material for: Triggers of acute attacks of gout, does age of gout onset matter? A primary care based cross-sectional study
Source: PLoS One. 2017 Oct 12;12(10):e0186096. doi: 10.1371/journal.pone.0186096 (PMC5638318; doi:10.1371/journal.pone.0186096)
Supplement: S1 File — (DOCX) [file pone.0186096.s002.docx]

**Supplementary material:**

In your experience are your gout attacks triggered by anything you do or don’t do (including diet, alcohol intake) in the previous one or two days? Yes No

If yes, please list them in the space provided:

________________________________________________________________________________________________________________________________________________________________________________________________________________________________________________________________________________________________________________________________________________________________________________________________________________________________________________________________________________________________________________________________________________________

In your experience are your gout attacks triggered by unduly warm weather? Yes No

In your experience are your gout attacks triggered by unduly cold weather? Yes No

Please think about the last gout attack you had, and answer the following:

- Did you undertake more than the normal amount of exercise on that day or on the previous day? Yes No
- Did you injure your joint in any way on that day or on the previous day?

Yes No

- Did you eat or drink alcohol excessively compared to your normal on that day or on the previous day? Yes No
- Did you have more than your usual portion of beef, pork or lamb compared to your normal on that day or on the previous day? Yes No
- Did you drink less water, tea, coffee, fizzy drink etc. on that day or on the previous day? Yes No
